# Professionals’ experience of the rapid implementation of a remote consultation model of healthcare: A survey of clinicians in the early stages of the COVID-19 pandemic

**DOI:** 10.1192/j.eurpsy.2021.284

**Published:** 2021-08-13

**Authors:** M. Boughdady, L. Connah, J. Inman, S. Jaydeokar, D. Marnoch, R. Nathan

**Affiliations:** 1 Inpatient Psychiatry, Chester and Wirral Partnership, BQ, United Kingdom; 2 Effective Services, Chester and Wirral Partnership, BQ, United Kingdom; 3 Community Learning Disability Team, Chester and Wirral Partnership, BQ, United Kingdom

**Keywords:** COVID-19, Technology

## Abstract

**Introduction:**

Despite the availability of remote consolation and the evidence for its effectiveness, its adoption has been relatively limited (Hashiguchi, 2020). In light of COVID social distancing measures, there was an immediate requirement to adopt this technology into routine practice.

**Objectives:**

The objective of this evaluation was to examine clinicians’ experiences of the urgent adoption of digital technology in a NHS provider of mental health and community physical health services.

**Methods:**

From a staff survey (n=234) of experiences of working during a period when there were significant levels of Covid-related restrictions, data was extracted and subject to thematic analysis by a research team made up of clinicians, academics, and quality improvement specialists.

**Results:**

Five key themes relevant to the urgent adoption of digital technology were identified (figure 1): (1) Availability of staff for patient contact was generally felt to be improved; (2) Quality of contact was reported to be variable (e.g. some respondents reporting better rapport with patients, whereas others found remote contact interfered with rapport building); (3) Safeguarding concerns were reported to be more difficult to identify through remote consultation; (4) Contingency plans were recommended to allow for vulnerable patients for whom remote consultation was a problem; (5) Multi-agency working was reported to be strengthened.

**Figure:**
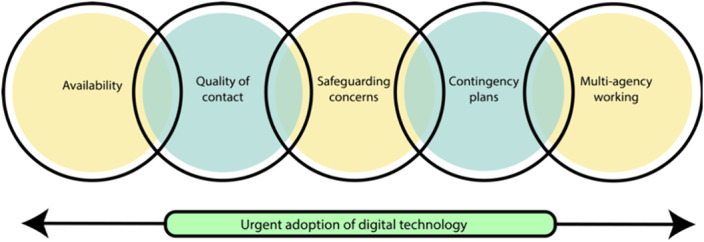

**Conclusions:**

The findings from this evaluation allow for an informed approach to future adoption of remote consultation in routine practice.

**Disclosure:**

No significant relationships.

